# Development and validation of a predicted nomogram for mortality of COVID-19: a multicenter retrospective cohort study of 4,711 cases in multiethnic

**DOI:** 10.3389/fmed.2023.1136129

**Published:** 2023-09-01

**Authors:** Yuchen Shi, Ze Zheng, Ping Wang, Yongxin Wu, Yanci Liu, Jinghua Liu

**Affiliations:** ^1^Center for Coronary Artery Disease (CCAD), Beijing Anzhen Hospital, Capital Medical University, and Beijing Institute of Heart, Lung and Blood Vessel Diseases, Beijing, China; ^2^Zhengzhou Central Hospital Affiliated to Zhengzhou University, Zhengzhou, Henan Province, China

**Keywords:** coronavirus disease 2019 (COVID-19), mortality, nomogram, prediction, LASSO

## Abstract

**Background:**

Coronavirus disease 2019 (COVID-19) is an infectious disease spreading rapidly worldwide. As it quickly spreads and can cause severe disease, early detection and treatment may reduce mortality. Therefore, the study aims to construct a risk model and a nomogram for predicting the mortality of COVID-19.

**Methods:**

The original data of this study were from the article “*Neurologic Syndromes Predict Higher In-Hospital Mortality in COVID-19.”* The database contained 4,711 multiethnic patients. In this secondary analysis, a statistical difference test was conducted for clinical demographics, clinical characteristics, and laboratory indexes. The least absolute shrinkage and selection operator (LASSO) and multivariate logistic regression analysis were applied to determine the independent predictors for the mortality of COVID-19. A nomogram was conducted and validated according to the independent predictors. The area under the curve (AUC), the calibration curve, and the decision curve analysis (DCA) were carried out to evaluate the nomogram.

**Results:**

The mortality of COVID-19 is 24.4%. LASSO and multivariate logistic regression analysis suggested that risk factors for age, PCT, glucose, D-dimer, CRP, troponin, BUN, LOS, MAP, AST, temperature, O_2_Sats, platelets, Asian, and stroke were independent predictors of CTO. Using these independent predictors, a nomogram was constructed with good discrimination (0.860 in the C index) and internal validation (0.8479 in the C index), respectively. The calibration curves and the DCA showed a high degree of reliability and precision for this clinical prediction model.

**Conclusion:**

An early warning model based on accessible variates from routine clinical tests to predict the mortality of COVID-19 were conducted. This nomogram can be conveniently used to facilitate identifying patients who might develop severe disease at an early stage of COVID-19. Further studies are warranted to validate the prognostic ability of the nomogram.

## 1. Introduction

Coronavirus disease 2019 (COVID-19), an infectious disease caused by the novel binuclear virus—severe acute respiratory syndrome coronavirus 2 (SARS-CoV-2), has been broken out and rapidly spread worldwide (1). The number of affected countries and deaths has risen dramatically, which is providing significant challenges and placing an unprecedented economic burden on global public health systems and clinical management (2).

COVID-19 can affect multisystem organs, and the main clinical presentation is pneumonia (3). Although most patients with COVID-19 have mild to moderate illness, with common respiratory symptoms and a good prognosis, several severe and critical patients will get worse rapidly with acute respiratory distress syndrome (ARDS), septic shock, multiple organ dysfunction, and even death, especially in those of the elderly with comorbidities such as congestive heart failure (CHF), chronic obstructive pulmonary disease (COPD), central nervous system (CNS) disease, chronic renal failure, and cancer (4). Furthermore, as previously described, deaths were more common in older patients with abnormal laboratory indexes such as inflammatory factors and hepatorenal function after COVID-19 affected (5). Therefore, it is crucial and urgent to rapidly identify prognostic indicators of fatal outcomes through efficient predictive methods to aid in the early implementation of preventive measures and interventions, thereby preventing disease progression and mortality in critically ill patients. The accurate and immediate decision-making of treatment strategies may reduce the mortality risk.

Considering radiological abnormalities were not observed during initial presentation in approximately 20% of cases, clinical characteristics and routine clinical laboratory tests may provide such prognostic factors as quickly as possible (6). The nomogram is a two-dimensional graphic mathematical representation of a scoring model made up of multiple scale axes designed for a user-friendly interface, highly accurate to calculate the probability of an outcome (7). A nomogram including variables like routine laboratory tests might be more effective and affordable for predicting the risk of mortality (8). Therefore, the purpose of this study was to describe the clinical features of COVID-19 and establish a nomogram based on a large number of COVID-19 patients incorporating common clinical demographics, characteristics, and laboratory parameters, to early warn the risk of fatal outcomes in patients with COVID-19.

## 2. Methods

### 2.1. Data source

The original database of this research was from the *Neurologic Syndromes Predict Higher In-Hospital Mortality in COVID-19* (9). Since Eskandar et al. had relinquished the ownership of the original database to *Neurology*,^1^ we can use this database to conduct secondary analysis according to different scientific hypotheses. The original research was granted exempt status. The requirement for obtaining informed consent was waived by the Ethics Committee for Clinical Research of the Albert Einstein College of Medicine, Montefiore Medical Center (9).

The original database collected consecutive hospitalized patients with moderate or severe COVID-19 from four hospitals in the Montefiore Health System between March 1st and April 16th, 2020. The database contained multiethnic patients, including 1743 Black, 466 White, 121 Asian, and 1753 Latino. The diagnosis of COVID-19 was based on World Health Organization interim guidance and confirmed by real-time reverse transcriptase PCR positive assay testing for SARS-CoV-2 RNA (10).

### 2.2. Study population and covariates

A total of 4,711 patients with confirmed COVID-19 were consecutively collected between March 1st and April 16th, 2020. We divided the whole participants into a derivation or validation cohorts by 7:3, randomly. The derivation cohort was formed of 3,534 subjects, including 2,661 surviving patients and 873 deceased patients. The validation cohort was formed of 1,177 subjects, including 902 surviving patients and 275 deceased patients.

Information on clinical demographics, characteristics, laboratory indexes, comorbidities, and mortality was collected by a health care surveillance software package (Clinical Looking Glass; Streamline Health, Atlanta, GA) and a review of the primary medical records (11).

### 2.3. Regression analysis

Least absolute shrinkage and selection operator (LASSO) regression analysis was applied to identify factors related to the mortality of patients with COVID-19. The Lambda values were chosen after a 10-fold cross-validation. Subsequently, a multivariate logistic regression analysis was established with the selection of LASSO regression analysis, in which *p*-value levels for inclusion criteria were conducted as 0.05.

### 2.4. Model development

Predictive models related to the mortality of COVID-19 were conducted in the primary cohort according to the variables selected by multivariate logistic regression analysis. The final model was determined by the Akaike information criterion (AIC), the receiver operating characteristic (ROC) curves, and the Harrell concordance index (C-index). The nomogram was derived from the final model.

### 2.5. Performance of the nomogram

The model was internally validated by data from the validation cohort. Discriminatory performance was measured by the C index. Calibration was tested via a calibration plot with 1,000 bootstraps resamples, which described the degree of fit between actual and nomogram-predicted mortality of COVID-19.

### 2.6. Clinical usage

Regarding its clinical usefulness, the decision curve analysis (DCA) was undertaken to assess the clinical benefit of the nomogram. Detailed descriptions of DCA have been previously reported (12). The results were considered statistically significant at *p*-value <0.05.

### 2.7. Statistical analysis

Continuous variables were expressed as medians (mean ± standard deviation), and categorical variables were expressed as numbers (percentage). Differences in baseline characteristics between groups for continuous variables were assessed by the Mann–Whitney U test. The Chi-square test or Fisher’s exact test was used for categorical variables according to their sample size.

A two-sided *p* < 0.05 was considered statistically significant. IBM SPSS v23.0 (SPSS Inc., Chicago, IL, United States) was applied for statistical analyses in the research. The nomogram was conducted and calibration curve analysis were carried out by the R software v4.2.0 (http://www.R-project.org, R Foundation for Statistical Computing, Vienna, Austria).

## 3. Results

### 3.1. Population clinical characteristics of the derivation and validation set

Table 1 summarized the clinical characteristics of the derivation set (*n* = 3,534) and the validation set (*n* = 1,177). The clinical demographics including mortality, race, comorbidities, and laboratory indexes did not have significant differences between both cohorts (all *p >* 0.05).

**Table 1:** Baseline clinical characteristics of the derivation and validation cohort.

Variable	Overall (*n* = 4,711)	Validation cohort (*n* = 1,177)	Derivation cohort (*n* = 3,534)	*p* value
Death, *n* (%)				0.354
No	3,563 (75.6)	902 (76.6)	2,661 (75.3)	
Yes	1,148 (24.4)	275 (23.4)	873 (24.7)	
Black, *n* (%)				0.190
No	2,968 (63.0)	708 (60.2)	2,260 (64)	
Yes	1743 (37.0)	469 (39.8)	1,274 (36)	
White, *n* (%)				0.606
No	4,245 (90.1)	1,056 (89.7)	3,189 (90.2)	
Yes	466 (9.9)	121 (10.3)	345 (9.8)	
Asian, *n* (%)				0.185
No	4,590 (97.4)	1,153 (98)	3,437 (97.3)	
Yes	121 (2.6)	24 (2)	97 (2.7)	
Latino, *n* (%)				0.005
No	2,958 (62.8)	779 (66.2)	2,179 (61.7)	
Yes	1753 (37.2)	398 (33.8)	1,355 (38.3)	
MI, *n* (%)				0.259
No	4,510 (95.7)	1,120 (95.2)	3,390 (95.9)	
Yes	201 (4.3)	57 (4.8)	144 (4.1)	
CHF, *n* (%)				0.250
No	4,170 (88.5)	1,063 (90.3)	3,107 (87.9)	
Yes	541 (11.5)	114 (9.7)	427 (12.1)	
CVD, *n* (%)				0.055
No	4,205 (89.3)	1,069 (90.8)	3,136 (88.7)	
Yes	506 (10.7)	108 (9.2)	398 (11.3)	
COPD, *n* (%)				0.639
No	4,446 (94.4)	1,114 (94.6)	3,332 (94.3)	
Yes	265 (5.6)	63 (5.4)	202 (5.7)	
DM.Complicated, *n* (%)				0.884
No	4,216 (89.5)	1,052 (89.4)	3,164 (89.5)	
Yes	495 (10.5)	125 (10.6)	370 (10.5)	
DM.Simple, *n* (%)				0.593
No	4,025 (85.4)	1,000 (85)	3,025 (85.6)	
Yes	686 (14.6)	177 (15)	509 (14.4)	
Renal.Disease, *n* (%)				0.421
No	3,878 (82.3)	978 (83.1)	2,900 (82.1)	
Yes	833 (17.7)	199 (16.9)	634 (17.9)	
All.CNS, *n* (%)				0.524
No	4,104 (87.1)	1,019 (86.6)	3,085 (87.3)	
Yes	607 (12.9)	158 (13.4)	449 (12.7)	
Stroke, *n* (%)				0.284
No	4,653 (98.8)	1,159 (98.5)	3,494 (98.9)	
Yes	58 (1.2)	18 (1.5)	40 (1.1)	
Age	63.370 ± 16.702	63.046 ± 16.556	63.477 ± 16.751	0.443
Temperature	37.318 ± 0.905	37.304 ± 0.844	37.323 ± 0.925	0.540
MAP	85.717 ± 16.477	85.182 ± 16.815	85.895 ± 16.362	0.199
O_2_Sats	92.149 ± 11.575	92.302 ± 11.674	92.099 ± 11.543	0.602
LOS	7.677 ± 6.780	7.680 ± 6.764	7.676 ± 6.786	0.987
WBC	8.638 ± 7.259	8.549 ± 9.493	8.667 ± 6.345	0.629
Lymphocytes	1.334 ± 4.860	1.486 ± 7.597	1.284 ± 3.503	0.217
Platelets	231.191 ± 111.035	226.768 ± 101.057	232.664 ± 114.139	0.115
Ferritin	1322.158 ± 3072.975	1330.418 ± 3362.145	1319.408 ± 2970.928	0.915
Glucose	179.073 ± 105.377	178.976 ± 106.158	179.105 ± 105.131	0.971
Sodium	138.089 ± 7.362	138.009 ± 7.253	138.115 ± 7.399	0.667
PCT	2.016 ± 6.371	1.925 ± 6.127	2.046 ± 6.451	0.574
CRP	11.814 ± 10.609	11.409 ± 10.497	11.949 ± 10.644	0.131
D-dimer	3.768 ± 5.150	3.617 ± 5.076	3.818 ± 5.174	0.246
AST	65.494 ± 204.217	61.038 ± 152.262	66.979 ± 218.798	0.387
ALT	44.293 ± 108.873	43.398 ± 113.252	44.591 ± 107.389	0.745
Troponin	0.056 ± 0.268	0.051 ± 0.196	0.058 ± 0.288	0.472
INR	1.192 ± 0.952	1.154 ± 0.809	1.204 ± 0.994	0.119
BUN	30.529 ± 30.546	29.911 ± 30.296	30.735 ± 30.630	0.423
Creatinine	1.988 ± 2.624	1.951 ± 2.560	2.000 ± 2.645	0.578

MI, myocardial infarction; CHF, congestive heart failure; CVD, cerebrovascular disease; COPD, chronic obstructive pulmonary disease; DM, diabetes mellitus; CNS, central nervous system; MAP, median arterial blood pressure; O2Sats, oxygen saturation; LOS, length of stay; WBC, white blood cells; PCT, procalcitonin; CRP, C-reactive protein; AST, aspartate aminotransferase; ALT, alanine aminotransferase; INR, international normalized ratio; BUN, blood urea nitrogen.

### 3.2. Comparison of the baseline characteristics of the derivation set between the survived patients and deceased patients

The baseline clinical characteristics of the derivation set were shown in Table 2. Compared with surviving patients, patients deceased were more likely to be white race and older, with comorbidities including CHF, COPD, renal disease, CNS disease, and stroke (all *p* < 0.05). Meanwhile, they were more frequently encountered with decreased median arterial blood pressure (MAP), oxygen saturation (O_2_Sats), and longer length of stay (LOS) (all *p* < 0.01). In the laboratory parameters between the two groups, the deceased patients showed significantly higher white blood cells (WBC), ferritin, glucose, sodium, procalcitonin (PCT), C-reactive protein (CRP), D-dimer, aspartate aminotransferase (AST), alanine aminotransferase (ALT), troponin, international normalized ratio (INR), blood urea nitrogen (BUN), and creatinine, but lower platelets (all *p* < 0.01).

**Table 2:** Clinical characteristics of the derivation cohort.

Variable	Overall	Death		*p* value
	(*n* = 3,534)	No (*n* = 2,661)	Yes (*n* = 873)	
Black, *n* (%)				0.232
No	2,260 (64.0)	1,687 (63.4)	573 (65.6)	
Yes	1,274 (36.0)	974 (36.6)	300 (34.4)	
White, *n* (%)				0.038
No	3,189 (90.2)	2,417 (90.8)	772 (88.4)	
Yes	345 (9.8)	244 (9.2)	101 (11.6)	
Asian, *n* (%)				0.335
No	3,437 (97.3)	2,592 (97.4)	845 (96.8)	
Yes	97 (2.7)	69 (2.6)	28 (3.2)	
Latino, *n* (%)				0.890
No	2,179 (61.7)	1,639 (61.6)	540 (61.9)	
Yes	1,355 (38.3)	1,022 (38.4)	333 (38.1)	
MI, *n* (%)				0.632
No	3,390 (95.9)	2,555 (96)	835 (95.6)	
Yes	144 (4.1)	106 (4)	38 (4.4)	
CHF, *n* (%)				0.048
No	3,107 (87.9)	2,356 (88.5)	751 (86)	
Yes	427 (12.1)	305 (11.5)	122 (14)	
CVD, *n* (%)				0.284
No	3,136 (88.7)	2,370 (89.1)	766 (87.7)	
Yes	398 (11.3)	291 (10.9)	107 (12.3)	
COPD, *n* (%)				0.011
No	3,332 (94.3)	2,524 (94.9)	808 (92.6)	
Yes	202 (5.7)	137 (5.1)	65 (7.4)	
DM.Complicated, *n* (%)				0.346
No	3,164 (89.5)	2,375 (89.3)	789 (90.4)	
Yes	370 (10.5)	286 (10.7)	84 (9.6)	
DM.Simple, *n* (%)				0.678
No	3,025 (85.6)	2,274 (85.5)	751 (86)	
Yes	509 (14.4)	387 (14.5)	122 (14)	
Renal.Disease, *n* (%)				0.023
No	2,900 (82.1)	2,206 (82.9)	694 (79.5)	
Yes	634 (17.9)	455 (17.1)	179 (20.5)	
All.CNS, *n* (%)				< 0.001
No	3,085 (87.3)	2,374 (89.2)	711 (81.4)	
Yes	449 (12.7)	287 (10.8)	162 (18.6)	
Stroke, *n* (%)				< 0.001
No	3,494 (98.9)	2,643 (99.3)	851 (97.5)	
Yes	40 (1.1)	18 (0.7)	22 (2.5)	
Age	63.477 ± 16.751	60.648 ± 16.799	72.101 ± 13.308	< 0.001
Temperature	37.323 ± 0.925	37.316 ± 0.872	37.345 ± 1.071	0.420
MAP	85.895 ± 16.362	89.312 ± 12.475	75.481 ± 21.579	< 0.001
O_2_Sats	92.099 ± 11.543	92.967 ± 11.189	89.453 ± 12.190	< 0.001
LOS	7.676 ± 6.786	7.294 ± 6.517	8.841 ± 7.430	< 0.001
WBC	8.667 ± 6.345	8.418 ± 6.323	9.428 ± 6.356	< 0.001
Lymphocytes	1.284 ± 3.503	1.338 ± 3.905	1.120 ± 1.778	0.112
Platelets	232.664 ± 114.139	237.181 ± 116.569	218.895 ± 105.265	< 0.001
Ferritin	1319.408 ± 2970.928	1082.743 ± 1557.485	2040.786 ± 5260.186	< 0.001
Glucose	179.105 ± 105.131	173.975 ± 103.171	194.742 ± 109.480	< 0.001
Sodium	138.115 ± 7.399	137.580 ± 6.636	139.746 ± 9.160	< 0.001
PCT	2.046 ± 6.451	1.340 ± 4.911	4.198 ± 9.426	< 0.001
CRP	11.949 ± 10.644	10.194 ± 9.430	17.297 ± 12.237	< 0.001
D-dimer	3.818 ± 5.174	3.133 ± 4.515	5.906 ± 6.361	< 0.001
AST	66.979 ± 218.798	56.350 ± 102.500	99.377 ± 400.644	< 0.001
ALT	44.591 ± 107.389	40.428 ± 54.930	57.283 ± 193.149	< 0.001
Troponin	0.058 ± 0.288	0.038 ± 0.106	0.118 ± 0.546	< 0.001
INR	1.204 ± 0.994	1.166 ± 0.926	1.322 ± 1.172	< 0.001
BUN	30.735 ± 30.630	26.239 ± 26.907	44.439 ± 36.647	< 0.001
Creatinine	2.000 ± 2.645	1.780 ± 2.454	2.670 ± 3.063	< 0.001

MI, myocardial infarction; CHF, congestive heart failure; CVD, cerebrovascular disease; COPD, chronic obstructive pulmonary disease; DM, diabetes mellitus; CNS, central nervous system; MAP, median arterial blood pressure; O2Sats, oxygen saturation; LOS, length of stay; WBC, white blood cells; PCT, procalcitonin; CRP, C-reactive protein; AST, aspartate aminotransferase; ALT, alanine aminotransferase; INR, international normalized ratio; BUN, blood urea nitrogen.

### 3.3. LASSO regression analysis

**Figure 1:**
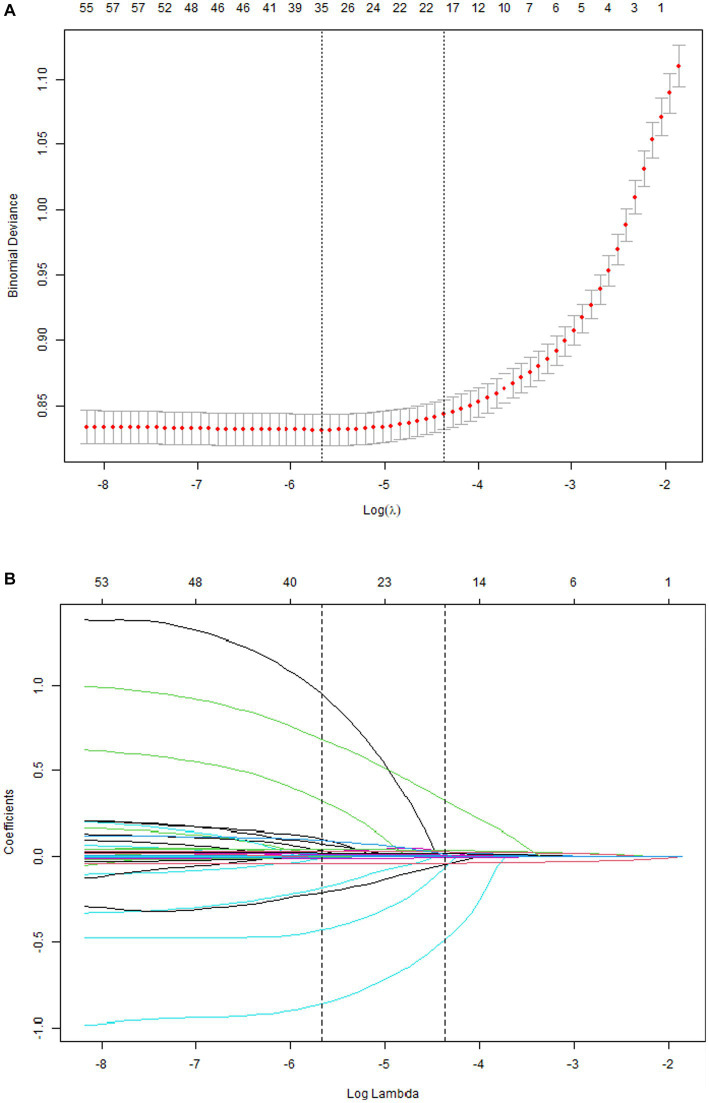
Risk factors selecting using LASSO model. 1A: Optimal parameter (lambda) selection for the LASSO model was cross-validated 6 the minimum criterion. Partial likelihood deviation curves (binomial deviation) versus log (lambda). The dotted vertical lines are drawn at the best values of 1SE (1-SE criterion) using the minimum criterion and the maximum criterion. 1B: LASSO coefficient profiles for 33 characteristics. The coefficient profiles were produced from logarithmic sequences (lambda). The vertical lines are drawn on the value selected using fivefold cross-validation, where the best lambda resulted in non-zero coefficients for five features.

A total of 33 related variables, including clinical demographics, laboratory indexes, and comorbidities, were initially put into the LASSO regression algorithm by 10-fold cross-validation to identify the indictors for mortality of COVID-19. As shown in Figure 1A, 16 potential indictors with non-zero coefficients were chosen: age, PCT, glucose, D-dimer, CRP, troponin, BUN, LOS, MAP, AST, temperature, O_2_Sats, platelets, Asian, CNS disease, and stroke. Figure 1B depicted the changes in the LASSO coefficients.

### 3.4. Multivariate logistic regression analysis

**Figure 2:**
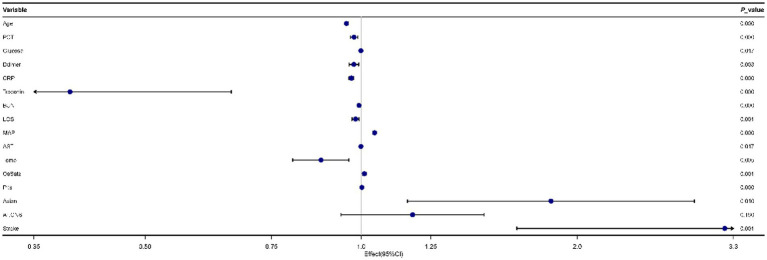
Forest plot for multivariate logistic regression analyses of predictors for mortality in patients with coronavirus disease 2019 (COVID-19).

As Figure 2 showed, 16 predictors chosen by the LASSO regression analysis were selected via multivariate logistic regression analysis to determine the independent parameters that predicted the mortality of COVID-19. 15 parameters were included in the final model, and those were age, PCT, glucose, D-dimer, CRP, troponin, BUN, LOS, MAP, AST, temperature, O_2_Sats, platelets, Asian, and stroke.

### 3.5. Construction of a novel nomogram scoring system

**Figure 3:**
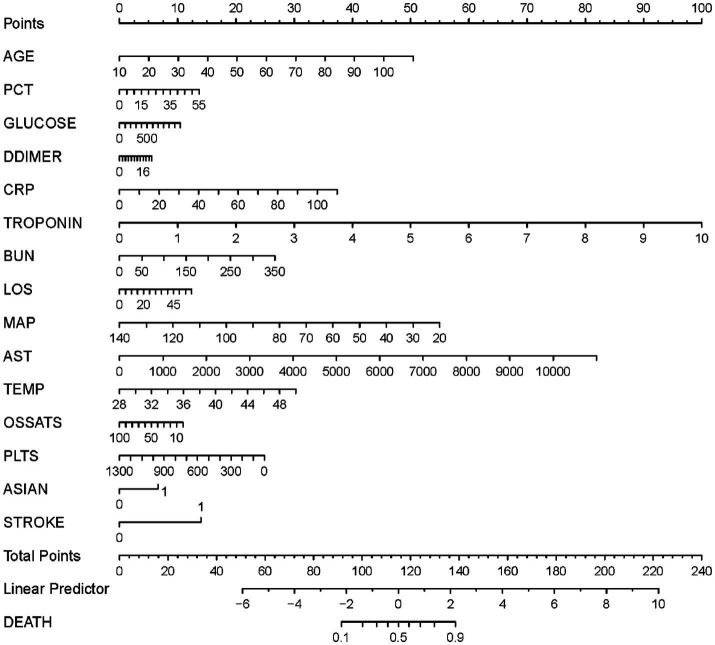
A nomogram prediction model for the probability of mortality in patients with coronavirus disease 2019 (COVID-19). PCT, procalcitonin; CRP, C-reactive protein; BUN, Blood urea nitrogen; LOS, length of stay; MAP, median arterial blood pressure; AST, aspartate aminotransferase; Temp, temperature; O_2_Sats, oxygen saturation; Plts, platelets.

Based on the results of the multivariate logistic regression analysis, the 15 variables above were included as predictors to establish a nomogram (Figure 3). Each predicters corresponded to a score, and the total score was mapped to the prediction axis of the diagnosis, which could reflect the risk factors for mortality of COVID-19. As an example, to better explain the nomogram, if the patient was 70 years old (30 points), PCT of 10 ng/mL (2 points), glucose of 300 mg/dL (3 points), D-dimer of 6 mg/L (2 points), CRP of 20 mg/L (7 points), troponin of 0.2 ng/mL (2 points), BUN of 50 mg/dL (4 points), LOS of 10 days (2 points), MAP of 70 mm Hg (32 points), AST of 1,000 U/L (7 points), temperature of 38°C (14 points), O_2_Sats of 90% (1 point), platelets of 300 k/mm^3^ (19 points), Asian of yes (7 points), and stroke of yes (14 points), the total points was 144 and the probability of death was estimated to be more than 90%.

### 3.6. Evaluation and validation of the nomogram

**Figure 4:**
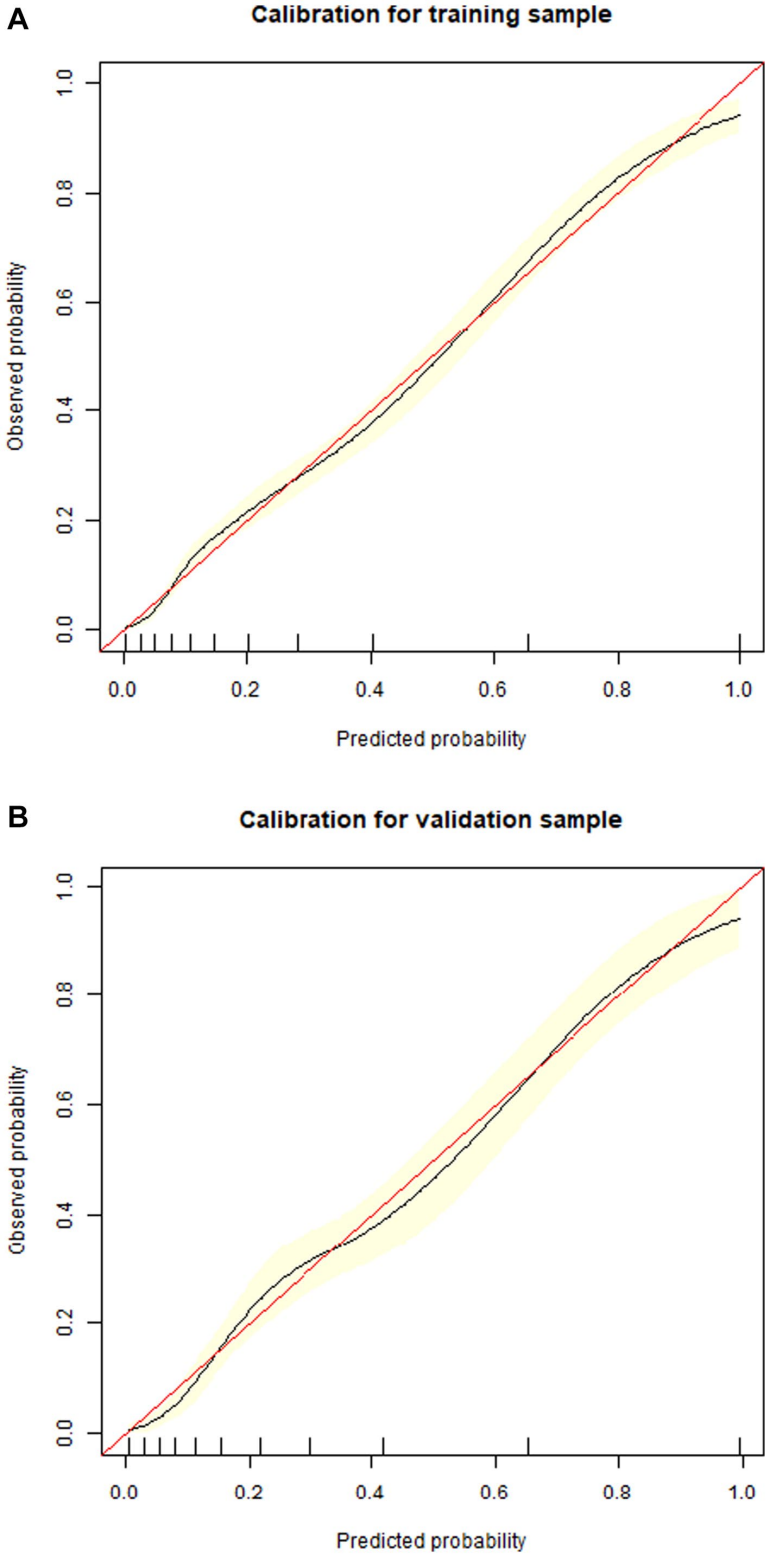
Calibration curves of the nomogram in the derivation cohort **(A)** and validation cohort **(B)**.

**Figure 5:**
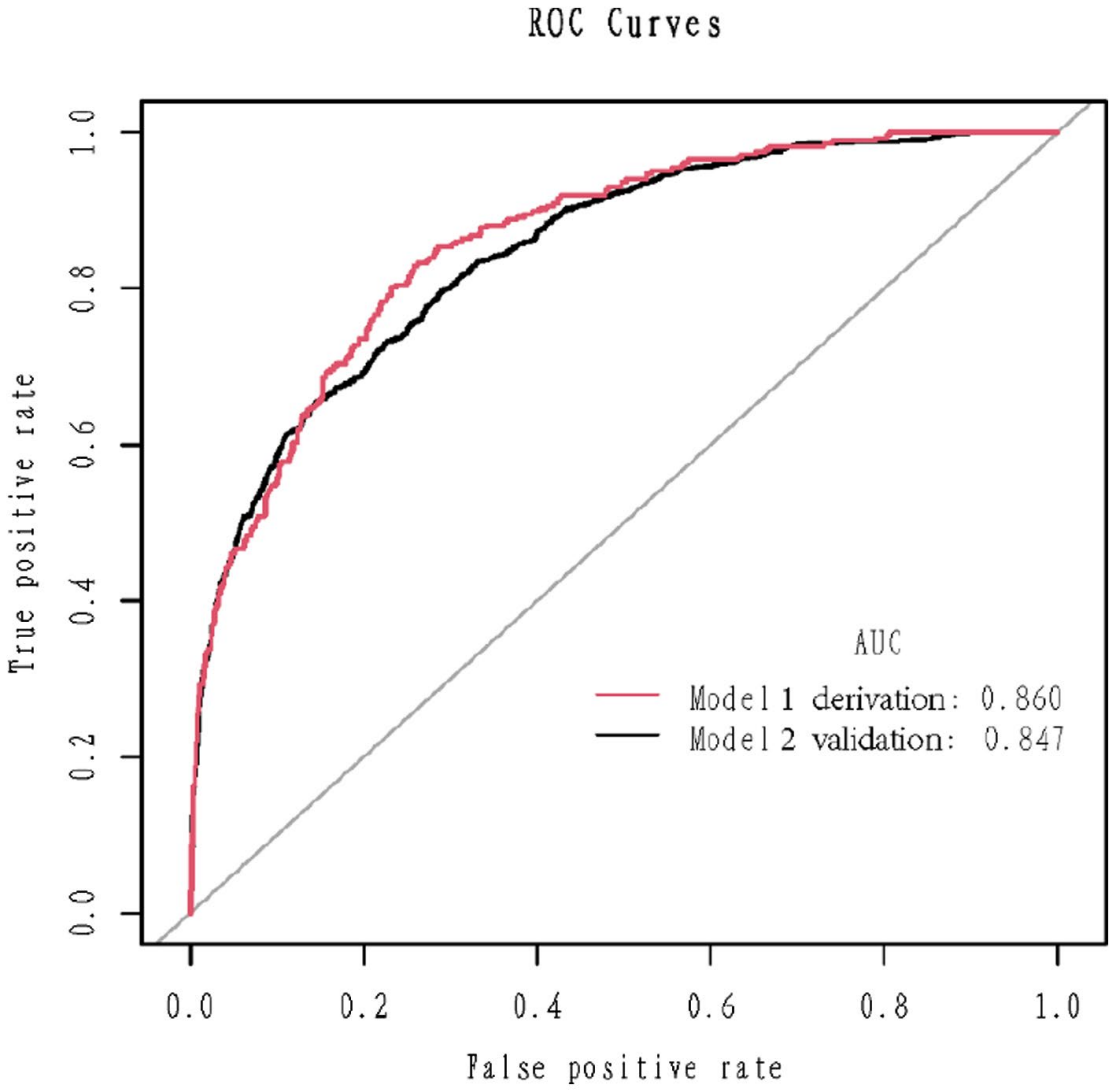
Receiver operating characteristics curve in the derivation cohort (red) and validation (blank) cohort for the nomogram.

**Figure 6:**
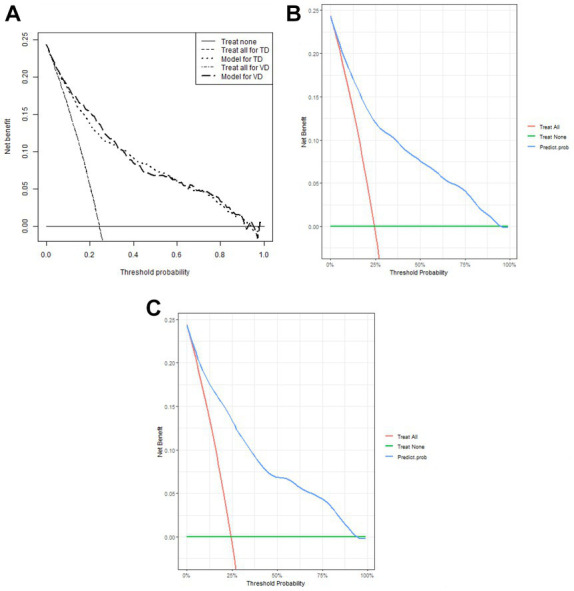
Decision curve analysis of the nomogram **(A)** and in the derivation cohort **(B)** and the validation cohort **(C)**, respectively.

1,177 patients constituted the validation cohort. The calibration curves were drawn to assess the model’s calibration in the derivation (Figure 4A) and validation cohort (Figure 4B). An analysis of the ROC curve was conducted to measure the discrimination of the model in the derivation and validation cohort. And the areas under the curves (AUC) were 0.860 and 0.847, respectively (Figure 5). In addition, the DCA curves showed that the novel nomogram also had a higher clinical net (Figure 6).

## 4. Discussion

In the current study, by employing a large, multicenter, and well-described population of 4,711 patient cohort, we used LASSO regression and multivariate logistic regression analysis to develop and validate a prediction nomogram. The nomogram was validated by internal 1,000 bootstrap resampling, as well as an internal validation cohort, maintaining an adequate calibration and discrimination capacity, which may enable physicians to predict the mortality of COVID-19 patients early and correctly for taking proactive measures accordingly.

In the research, clinical demographics, characteristics, and laboratory tests were collected and analyzed to investigate the risk of fatal outcomes in COVID-19 patients. Compared with other diseases, COVID-19 progresses more severely and faster, which may not be identified promptly (13). The early symptoms of COVID-19 are insidious and flexible, which creates more challenges to early detection (14). Especially during this time of the severe COVID-19 epidemic, many non-respiratory physicians involved in this critical battle for fighting against the epidemic, a more straightforward method that does not require professional respiratory doctors and radiologists to evaluate the infiltration of multiple lung lobes is practical (15). To make the prognostic nomogram rapid and easy to use in the busy clinical work, we only focused on variables in clinical features and laboratory tests.

The mortality of COVID-19 in our study is 24.4%, in line with the range reported in recent studies (16). In the nomogram, age was one of the most imperative predictors for mortality of COVID-19. Meanwhile, the deceased group was older compared to the surviving group. Previously, researches also reported elders in early risk evaluation for severe COVID-19 (17, 18). The association between age and severe COVID-19 might be related to angiotensin converting enzyme-2 (ACE2) (19). As previously described, ACE2 has essential salutary functions and could decrease several detrimental effects, such as inflammation, vasoconstriction, and thrombosis. However, SARS-CoV-2 can markedly downregulate ACE2 by entering into cells, which might be extra detrimental in the old population via age-related baseline ACE2 deficiency (20). In contrast, there were also several studies that suggested age was not an independent indicator for mortality or severe COVID-19 disease (21). As mentioned in those studies, the reason age was not an independent indicator might be the fact that, rather than age, age-related comorbidities affect mortality (22).

As for inflammation-related factors, including PCT, CRP, and D-dimer they were revealed to be related to the mortality of COVID-19 in our model, which was coincided with previous research (23). PCT and CRP are common inflammation factors in infected diseases (24). D-dimer is not only a fibrinogen-related factor, but also a thromboinflammatory factor (25). A high prevalence of pulmonary embolism and venous thromboembolism had been reported in patients with COVID-19 (26). Moreover, more than macrovascular thrombosis, microthrombotic events in the lungs have been observed by autopsies (27). A thromboinflammatory procedure in the pulmonary capillary vessels might be the major reason for microthrombosis in the lung capillaries, inducing COVID-19-associated coagulation disorder, which is characterized by a raising in procoagulant biomarkers, such as fibrinogen, together with a substantial rise in D-dimer (28).

For other laboratory parameters in the nomogram, blood glucose, BUN, troponin, and AST, which indicated multi-organ dysfunction, represented major predictors of mortality for COVID-19 patients (29). A Chinese meta-analysis demonstrated that diabetes mellitus was related to an increased risk of severity or death in COVID-19 patients, while it was still not clear to what extent diabetes mellitus independently contributed to the increased risk (30). In our model, not the diabetes condition but the glucose level contributed to the mortality of COVID-19. Therefore, controlling the glucose level is vital for neither diabetes or non-diabetes patients. In other nomogram models, high direct bilirubin level was confirmed to be an independent indicator of mortality in COVID-19 (31). Whereas, another research with a larger population revealed that rather than bilirubin, AST elevation was more closely related to COVID-19 mortality risk (32). SARS-CoV-2 mainly attacks the respiratory system. Moreover, previous research has shown evidence of damage to other organs, such as the liver (33). Liver dysfunction in COVID-19 patients might be mainly related to an organ-specific immune response (34). Also, systemic cytokine storm, hypoxemia, and medications can aggravate it (35). It was found that AST was increased in the severe COVID-19 patients (36). This result was coincided with the findings of our research, which indicated that AST was a critical biomarker for clinical outcomes. Besides, recent a study pointed out that acute kidney injury was closely related to severe infection and fatality in COVID-19 patients (37). Additionally, the combination of BUN and D-dimer could predict mortality in 305 COVID-19 patients, with 27.9% mortality (38).

It is noteworthy that, in our model, the race of Asian is a risk score for mortality of COVID-19. Even if we have not observed significant differences in mortality within our Asian population because there were only 97 patients in this database, it was included in the nomogram. Hoping larger datasets with more Asian people will improve the prediction model for our Asian.

In our research, a practical nomogram based on easily accessible variates from routine clinical work, can provide a more accurate evaluation and prediction of mortality for COVID-19 patients. As a result, clinicians can use this intuitive predictive nomogram to draw a few lines promptly to make a prompt calculation of a patient’s prognosis. If a patient with a high mortality rate could be identified properly and rapidly, he or she could be more likely to benefit from close attention in clinical care and nutritional support in nursing care, which would ultimately have a positive effect on recovery. In addition, our model could help doctors rationally allocate medical resources to reduce the mortality of COVID-19 when medical resources are scarce.

## 5. Limitations

Our study has several limitations. First of all, we found that the race of Asian might be an independent risk factor for the mortality of COVID-19. However, due to the Asian population in this database being too small, further research with larger Asian population are warranted to validate the finding. Second, the model verification method only used internal verification. More extensive studies and external validation should be needed to verify the nomogram.

## 6. Conclusion

In conclusion, we developed an early warning model based on accessible variates from routine clinical tests to predict the mortality of COVID-19. This nomogram could be conveniently used to facilitate identifying patients who might develop to severe disease at an early stage of COVID-19. Further researches are warranted to validate the prognostic ability of the nomogram.

## Data availability statement

The datasets presented in this study can be found in online repositories. The names of the repository/repositories and accession number(s) can be found at: https://doi.org/10.5061/dryad.7d7wm37sz.

## Ethics statement

The studies involving human participants were reviewed and approved by Ethics Committee for Clinical Research of the Albert Einstein College of Medicine, Montefiore Medical Center. The patients/participants provided their written informed consent to participate in this study.

## Author contributions

YS and JL conceived the study and designed the protocol. YS and ZZ integrated the data and wrote the manuscript. YW, PW, and YL were responsible for the selection of the study, the extraction of data, and the evaluation of the quality of the study. JL critically reviewed the manuscript. All authors contributed to the article and approved the submitted version.

## Funding

National Natural Science Fund of China (nos. 82200441, 81970291 and 82170344); Beijing Hospitals Authority Youth Programme (no. QML20230607); Young Elite Scientists Sponsorship Program by BAST (no. BYESS2023238); and the Major State Basic Research Development Program of China (973 Program, no. 2015CB554404) supported this work.

## Conflict of interest

The authors declare that the research was conducted in the absence of any commercial or financial relationships that could be construed as a potential conflict of interest.

## Publisher’s note

All claims expressed in this article are solely those of the authors and do not necessarily represent those of their affiliated organizations, or those of the publisher, the editors and the reviewers. Any product that may be evaluated in this article, or claim that may be made by its manufacturer, is not guaranteed or endorsed by the publisher.

## Footnotes

^1^ doi.org/10.5061/dryad.7d7wm37sz
